# What factors potentially influence the ability of phylogenetic distance to predict trait dispersion in a temperate forest?

**DOI:** 10.1002/ece3.3691

**Published:** 2017-12-20

**Authors:** Feng Jiang, Yanhan Xun, Huiying Cai, Guangze Jin

**Affiliations:** ^1^ Center for Ecological Research Northeast Forestry University Harbin China

**Keywords:** community assembly, functional trait, phylogenetic dispersion, phylogenetic diversity, phylogenetic signal, spatial scales, species pool

## Abstract

Although phylogenetic‐based approaches have been frequently used to infer ecological processes, they have been increasingly criticized in recent years. To date, the factors that affect phylogenetic signals and further the ability of phylogenetic distance to predict trait dispersion have been assumed but not empirically tested. Therefore, we investigate which factors potentially influence the ability of phylogenetic distance to predict trait dispersion. We quantified the phylogenetic and trait dispersions across size classes and spatial scales in a 9‐ha old‐growth temperate forest dynamics plot in northeastern China. Phylogenetic signals at the community level were generally lower than those at the species pool level, and phylogenetically clustered communities showed lower phylogenetic signals than did overdispersed communities. This pattern might explain the other three findings of our study. First, phylogenetically overdispersed communities performed better at predicting trait dispersion than did clustered communities. Second, the mean pairwise distance (MPD)‐based metric exhibited a stronger correlation with trait dispersion than did the mean nearest taxon distance (MNTD)‐based metric. Finally, the MNTD‐based metric showed that the prediction accuracy for trait dispersion decreased with increasing spatial scales, whereas its effects were weak on the MPD‐based metric. In addition, phylogeny could not determine the dispersions of all functional axes but was able to predict certain traits depending on whether they were evolutionarily conserved. These results were conserved when we removed the effects of space and environment. Our findings highlighted that using phylogenetic distance as a proxy of trait similarity might work in a temperate forest depending on the species in local communities sampled from total pool as well as the traits measured. Utilizing these rules, we should rethink the conclusions of previous studies that were based on phylogenetic‐based approaches.

## 1 INTRODUCTION

Seeking the mechanisms by which the diversity of life on Earth has been constructed, maintained, and recovered has long been one of the ultimate goals of ecologists. In the past few decades, two processes have been proposed for interpreting existing patterns in observed communities. The concept of niche has been used frequently to represent deterministic mechanisms, including abiotic and biotic interactions, which can shape species distribution and community assemblages (Chase & Leibold, 2003; Keddy, 1992; Tilman, 1982). The other mechanism is related to the neutral theory, which emphasizes the role of stochasticity and dispersal on community assembly (Hubbell, 1979, 2001). Recently, the test and discrimination between these ecological processes have benefited from phylogenetic‐based approaches (Webb, 2000; Webb, Ackerly, McPeek, & Donoghue, 2002). This approach suggests that ecological processes can be mirrored by the phylogenetic patterns that underlie the theoretical basis that overdispersed patterns imply biotic interaction and clustered patterns indicate abiotic filtering when the evolution of relevant traits is conserved (Kraft, Cornwell, Webb, & Ackerly, 2007; Webb et al., 2002). Since 2000, this approach has been used across a wide range of subjects (e.g., Cavender‐Bares, Kozak, Fine, & Kembel, 2009; Vamosi, Heard, Vamosi, & Webb, 2009) to compensate for the lack of a plant functional trait dataset.

Along with the pervasive application of this method in recent years, critiques from ecologists have also increased (Gerhold et al., 2015; Liu, Swenson, Zhang, Ma, & Thompson, 2013; Mayfield & Levine, 2010; Pavoine, Gasc, Bonsall, Mason, & Prinzing, 2013; Srivastava, Cadotte, MacDonald, Marushia, & Mirotchnick, 2012; Swenson, 2013). Specifically, Gerhold et al. (2015) critiqued seven potential assumptions of the phylogenetic‐based approaches. Liu et al. (2013) empirically tested that it was difficult for phylogeny to represent trait dispersion in a subtropical forest. Indeed, the conserved evolution of traits is a foundational assumption of the phylogenetic‐based approaches; however, the situation in a real community is usually different. Many studies showed that there were weak or no phylogenetic signals in traits (reviewed by Losos, 2008). Moreover, it is difficult for all trait sets to show phylogenetic signals, although some traits may show them (Swenson, Erickson, et al. 2012; Yang et al., 2014). These results have discouraged the wider utilization of phylogenetic‐based approaches (Gerhold et al., 2015; Swenson, 2013).

Recently, Cadotte, Davies, & Peres‐Neto (2017) gave a comprehensive discussion about why we might not find a relationship between phylogenetic distance and ecological differences. They suggested three ecological and evolutionary reasons and four shortcomings of experimental design and analysis, including intraspecific trait variation, tempo of evolution, complicated ecological processes, inappropriate species pool, lack of evolutionary models, and issues in experimental studies. For example, the species pool that is chosen can vastly influence the phylogenetic structure and the ability of phylogenetic distance to predict ecological differences (Cadotte et al., 2017; Swenson, Enquist, Jason, Jill, & Zimmerman, 2006). This issue was also discussed by Losos (2008) and was termed phylogenetic scale dependency. For example, in a Floridian plant community, the phylogenetic signal was higher when more clades were included with increasing spatial scales (Cavender‐Bares, Keen, & Miles, 2006). Silvertown, Dodd, Gowing, Lawson, and McConway (2006) proposed a hierarchical filtering model that suggested that α‐niche was more labile and β‐niche was more conserved for species coexistence. These are important guidelines for phylogenetic‐based studies that usually test phylogenetic signals at the species pool level only, in which the signals likely exist, but not at the community level where fewer clades are included (Srivastava et al., 2012). This problem may confound our understanding of observed patterns. Although misunderstanding due to the scale dependency of phylogenetic signals has been identified in recent theoretical reviews (Gerhold et al., 2015; Srivastava et al., 2012), their suggestions remain to be tested.

Inspired by recent theoretical reviews (Cadotte et al., 2017; Gerhold et al., 2015; Srivastava et al., 2012), we evaluate which factors influence the ability of phylogenetic distance to determine trait dispersion for a practical objective on the application of the phylogenetic‐based approaches in a temperate forest in northeastern China. We propose several predictions from the perspective of phylogenetic scale dependence that phylogenetic signal may decrease when fewer clades are included or among closely related species. First, a clustered phylogenetic pattern may weakly reflect trait dispersion because closer taxa are expected to be dispersed in the local community. Second, mean nearest taxon distance (MNTD), which depicts the dispersion of terminal taxa on a tree, may be a weaker predictor for trait dispersion than pairwise phylogenetic distance (MPD), which quantifies the overall dispersion of taxa on a tree (Webb et al., 2002). Third, based on the second prediction described above, as the spatial scale increases, a larger proportion of the species in the species pool are included, which may increase the relationship between the phylogenetic and trait dispersions for MPD. By contrast, with an increase in species at a broader spatial scale, a species has a higher probability of co‐occurring with their closely related species. Therefore, there may be a decreasing relationship between phylogenetic distance and trait dispersion for MNTD. In addition to the predictions above, the plant functional traits across different organs may be independent from one other (functional dimensionality; Laughlin & Wilson, 2014), which suggests that traits from contrasting functional axes may show inconsistent phylogenetic signals. This lead to our fourth prediction that phylogenetic distance is difficult or impossible to be used as a proxy for all independent trait dispersion patterns (theoretical reviews from Swenson, 2013; Gerhold et al., 2015).

Overall, we aim to evaluate the differences in the phylogenetic signals at the species pool and community levels and to identify the factors that influence the ability of phylogenetic distance to predict trait dispersion in a 9‐ha old‐growth temperate forest dynamics plot (FDP), which is an ideal platform because it is one of the best‐conserved communities in northeastern China. This research aims to (1) identify differences between phylogenetic signals at the species pool and community levels and (2) identify how phylogenetic pattern, phylogenetic metric, spatial scale, and functional dimensionality influence the ability of phylogenetic distance to predict trait dispersion.

## 2 MATERIALS AND METHODS

### 2.1 Study site

**Figure 1:**
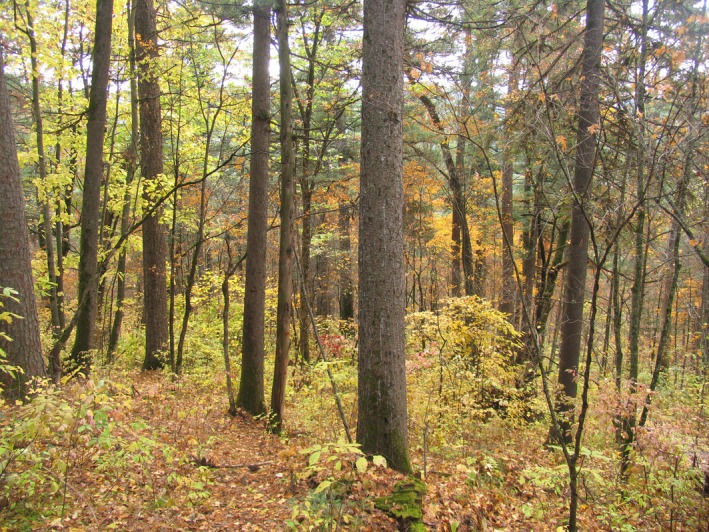
Our study was conducted in this old‐growth mixed broadleaved‐Korean pine forest, which was dominated by two abundant pine species, *Pinus koraiensis* and *Abies nephrolepis*, and accompanied by some deciduous species (e.g., *Corylus mandshurica* and *Acer mono*)

This study was conducted in a 9‐ha (300 m × 300 m) FDP located in the Liangshui National Reserve (47°10′50″N, 128°53′20″E) in northeastern China. The mean annual temperature in this region is −0.3°C, and the mean annual precipitation is 676 mm. This region is covered by snow for 130–150 days every year. We established the plot in 2005 following the Barro Colorado Island (BCI) plot protocol (Condit, 1998). We recensused this FDP for the first time in 2010 and documented 21,355 woody individuals ≥1 cm diameter at breast height (dbh; 1.3 m; 34,021 free‐standing stems) belonging to 48 species, 34 genera, and 20 families. Two abundant pine species, *Pinus koraiensis* and *Abies nephrolepis*, accompanied by some deciduous species (e.g., *Corylus mandshurica* and *Acer mono*) resulted in the old‐growth mixed broadleaved‐Korean pine forest type (Figure 1). The elevation ranged from 425 to 508 m. This study covered 41 of the 48 total species in our FDP, which accounted for 97.9% of all individuals.

### 2.2 Functional trait collection

We measured and compiled a total of eight functional traits, including woody density (WD), seed mass (SM), maximum height (*H*_max_), specific leaf area (SLA), leaf area (LA), leaf thickness (LT), leaf dry matter content (LDMC), and leaf phosphorus content (LPC), which represented the major axes of the plant function strategy (Swenson, Erickson, et al. 2012). WD was the oven‐dried mass (103°C, 72 hr) divided by the fresh volume measured by water displacement. Three to five individuals were collected near our FDP. For canopy species (dbh > 10 cm), WD was estimated as the annulus‐weighted average, where increment cores were divided into 1‐cm segments. For shrubs, the trunk was cut off directly. The SM was compiled from the Seeds of the Woody Plants in China (State Forestry Administration 2001). The *H*_max_ was compiled from the Flora of China (Wu & Raven, 1994–2009). Leaf traits were measured following a standard protocol (Pérez‐Harguindeguy et al., 2013). If available, five healthy adult individuals per species that were adequately exposed to sun were selected, and 20 leaves were collected from each individual (Jiang, Xun, Cai, & Jin, 2017). The SLA was the area of a leaf divided by the oven‐dried mass. The LA was the projected area of one side of a leaf. The LT was measured as the average thickness of a leaf on three loci. The LDMC was calculated as the oven‐dried mass of a leaf divided by its water‐saturated fresh mass. The LPC was the total amount of *P* per unit of dry leaf mass.

### 2.3 Phylogenetic reconstruction

To reduce the bias in the measurements due to the polytomies among the terminal taxa reconstructed by Phylomatic (Webb & Donoghue, 2005), particularly for MNTD, we reconstructed the phylogenetic relationship using DNA barcoding (Kress et al., 2009). Three sequences for each species were collected from GenBank (two plastid DNA genes for *rbcL* and *matK* and one nuclear DNA gene for *ITS*; some species were also sequenced following Kress et al. (2009), see the methods in Liu et al. (2013)). Only one of the 41 species did not have any of the three sequences, and we used the *rbcL* sequence from a congeneric species as a proxy. All sequences were aligned using MUSCLE (Edgar, 2004). We aligned the sequences of *rbcL* and *matK* globally, and we aligned the *ITS* sequences within orders or families. We combined the aligned *rbcL*,* matK*, and multiple *ITS* sequences into a supermatrix using the supermat function of the phylotools package implemented in R‐3.2.5 (R Core Team 2016). This supermatrix was then input into raxmlGUI version 1.5b1 (Silvestro & Michalak, 2011) to construct a maximum likelihood phylogeny. To maintain the topology coincident with the APG III phylogeny, we used an order‐level constraint tree constructed by Phylomatic to retain deep nodes a priori (Kress et al., 2010; Muscarella et al., 2014). This maximum likelihood tree was calibrated by nonparametric rate smoothing in the software r8s (Sanderson, 2003) to obtain an ultrametric phylogenetic tree (Figure S1), which was further used to calculate the phylogenetic dispersion.

### 2.4 Phylogenetic and trait analyses

We used the observed MPD and MNTD (Webb et al., 2002) to quantify the phylogenetic dispersion and make a comparison with the null communities. We generated 999 null communities via randomization of species names on the phylogenetic tree, and then we calculated the standardized effect size of MPD and MNTD (i.e., SES.MPD and SES.MNTD). The calculation was as follows:

$$\text{SES.MPD}=\left({\text{MPD}}_{\mathrm{obs}}-\text{mean}\left({\text{MPD}}_{\mathrm{null}}\right)\right)/SD\left({\text{MPD}}_{\mathrm{null}}\right)$$

$$\text{SES.MNTD}=\left({\text{MNTD}}_{\mathrm{obs}}-\text{mean}\left({\text{MNTD}}_{\mathrm{null}}\right)\right)/SD\left({\text{MNTD}}_{\mathrm{null}}\right)$$

where MPD and MNTD are the mean pairwise phylogenetic distance and the mean nearest taxon distance between all individuals within an observed (i.e., MPD_obs_ and MNTD_obs_) or random community (i.e., MPD_null_ and MNTD_null_), respectively. While the negative SES.MPD and SES.MNTD values represented phylogenetic clustering, the positive values indicated phylogenetic overdispersion. These analyses were repeated at multiple spatial scales (10 m × 10 m, 20 m × 20 m, 30 m × 30 m and 50 m × 50 m) and size classes (small, medium, and large). The size classes of the canopy species were divided into three stages: small (dbh ≤ 5.0 cm), medium (5.0 < dbh ≤ 10.0 cm), and large (dbh > 10.0 cm; Piao, Comita, Jin, & Kim, 2013). We regarded the species that had a maximum dbh that reached the maximum size class in our study (i.e., 10.0 cm) as the canopy species (28 species in total; Swenson, Enquist, Thompson, & Zimmerman, 2007; Yang et al., 2014). In addition, we performed these analyses for all 41 species (i.e., all individuals) for comparative analyses.

For simplification, we quantified trait dispersion using the same metrics and formula as phylogenetic dispersion (i.e., SES.MPD and SES.MNTD). The trait dendrograms were constructed for all eight traits and the eight individual traits to quantify trait dispersions for a comparison with the phylogenetic results (Swenson, Erickson, et al. 2012). To reduce trait redundancy when analyzing all traits, we first calculated the principal components (PCs) for all species and canopy species. Then, we chose the first five PCs (which explained 94.2% of the variation) for all species and the first four PCs (which explained 91.6% of the variation) for the canopy species to calculate the trait Euclidean distance matrix. Finally, dendrograms for all traits and for each of the eight individual traits were generated by performing hierarchical clustering. Prior to these analyses, all traits were log‐transformed and scaled to approximately a mean of zero with unit variance (Swenson, 2014). The trait dispersion was then quantified following the same steps as the computation of SES.MPD and SES.MNTD, using a trait dendrogram instead of phylogenetic tree.

Phylogenetic signal tests at the species pool level and the community level were implemented using Blomberg's *K* statistic (Blomberg, Garland, Ives, & Crespi, 2003). At the species pool level, all 41 species for all trees and 28 canopy species for three size classes (i.e., small, medium, and large) were used to test phylogenetic signals. At the community level, species occurring in a community were pruned from the phylogenetic tree of the species pool to generate a specific community‐level phylogenetic tree; this pruned tree was used to test phylogenetic signals, and the process was performed in all subcommunities in the FDP. We selected SLA, LA, and LT to compare the phylogenetic signals between the species pool level and the community level, respectively, because they showed stronger phylogenetic signals at the species pool level (*K *>* *1; Table 1). In addition, we divided all communities into phylogenetically clustered and overdispersed communities based on SES.MPD; then, we compared the difference of phylogenetic signals within communities with the different dispersion patterns. To test the significance of the *K* values, we randomly shuffled the trait data on the phylogenetic tree 999 times to generate null distributions and calculate *P* values (Swenson, 2014). These tests were implemented using the multiPhylosignal function of the picante package.

**Table 1:** Phylogenetic signal tests using Blomberg's *K* statistic at the species pool level

Traits	No. of species	*K*	*P* value
Woody density	41/28	0.309/0.360	.056/.058
Seed mass	41/28	0.180/0.271	.675/.230
Maximum height	41/28	0.418/0.604	.008/.004
Specific leaf area	41/28	1.102/1.302	<.001/<.001
Leaf area	41/28	2.351/2.441	<.001/<.001
Leaf thickness	41/28	1.884/2.819	<.001/<.001
Leaf dry matter content	41/28	0.542/0.400	<.001/.048
Leaf phosphorus content	41/28	0.232/0.308	.203/.114

The left values of “/” indicate all 41 species; the right values indicate 28 canopy species (dbh > 10 cm). All traits were log‐transformed.

### 2.5 Controlling for environmental and spatial effects

In addition to phylogeny, abiotic variables and dispersal limitation may also play important roles in trait dispersion due to the resulting autocorrelation among communities (Legendre et al., 2009; Liu et al., 2013). Therefore, another analysis was essential to control for their effects on trait dispersion. This analysis was implemented via the distance‐based Moran's eigenvector maps (dbMEM) and the variation partitioning approach (Borcard & Legendre, 2002; Dray, Legendre, & Peres‐Neto, 2006; Peres‐Neto, Leibold, & Dray, 2012). Abiotic variables included topographical and soil factors. Specifically, topography was a proxy for relevant environmental variables we did not measure. Topography involved mean elevation, convexity, slope, and aspect in the FDP, which were calculated based on elevation (see detailed descriptions in Legendre et al., 2009 and De Cáceres et al., 2012). Ten variables were measured to describe the soil environment in our FDP (pH, total N, total P, available K, available N, available P, organic C, bulk density, soil moisture, and mass water content). We used a combination of systematic and random sampling approaches to collect soil subsamples based on the 20‐m grid within our plot (a point at the intersection from which two additional points were selected in a random direction [N, NE, E, SE, S, SW, W, or NW] and distance [2, 5 or 8 m]; Shi, Gao, Cai, & Jin, 2015). Soil and topographical variables (except for aspect) were used to construct third‐degree polynomial equations coupled with sin(aspect) and cos(aspect) variables to generate a total of 41 variables.

Dispersal limitation was coarsely and simply represented by spatial variables in our study. Spatial variables were generated by dbMEM‐based eigenvectors for all four spatial scales. A distance matrix among subplots was generated using the Euclidean distance between the center points of the cells based on their coordinates. This matrix was then truncated to retain the appropriate values and replaced four times with other values (Legendre & Legendre, 2012). Eigenvectors corresponding to positive eigenvalues were generated by implementing a principal coordinate analysis (PCoA) on the truncated distance matrix.

Two analyses were implemented in our study. First, we assessed the ability of phylogenetic distance to predict trait dispersion by controlling for only the spatial variables (i.e., eigenvectors); second, we controlled for both abiotic and spatial variables. Forward selection procedures (Blanchet, Legendre & Borcard 2008) were implemented on environmental and spatial variables to generate variables that significantly correlated with trait dispersion. These retained variables (matrix 1) were combined with phylogenetic dispersion (matrix 2) for variation partitioning analysis using the varpart function in the vegan package. The forward selection procedures were performed via the forward.sel function with 9,999 permutations in the packfor package, and significance tests for the pure phylogenetic dispersion effect were performed with the anova.cca function in the vegan package with 9,999 permutations (Liu et al., 2013).

## 3 RESULTS

### 3.1 Phylogenetic signal tests and phylogenetic, trait dispersions

**Figure 2:**
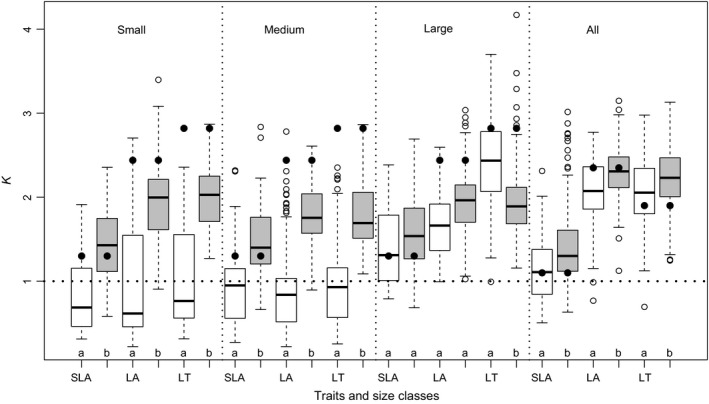
Phylogenetic signal tests (Blomberg's *K* values) of specific leaf area (SLA), leaf area (LA), and leaf thickness (LT) at the species pool and community levels at the 20 m × 20 m spatial scales. “small” represents small size class communities, “medium” represents medium size class communities, “large” indicates large size class communities, and “all” indicates a mix of all trees. The white boxes indicate *K* values of clustered phylogenetic dispersions followed by its paired black boxes representing *K* values of overdispersed phylogenetic dispersions. The black closed dots in each box display the *K* values at the species pool level. The division of phylogenetic pattern is based on SES.MPD. T tests was performed between paired clustered and overdispersed patterns for each trait at each size class (a‐a, nonsignificance; a‐b, significance)

Inconsistent phylogenetic signals were found at both the species pool and community levels. At the species pool level, LA, LT, SLA, LDMC, and *H*_max_ showed significant phylogenetic signals (Table 1). The *K* values of LA, LT, and SLA were greater than one, which implied more conserved evolution than the Brownian model. At the community level, the phylogenetic signals for LA, LT, and SLA were generally lower than those at the species pool level (Figure 2). When analyzing different phylogenetic patterns separately, we found clustered communities showed lower *K* values than overdispersed communities, which were closer to or exceeded the *K* values at the species pool level (e.g., SLA).

Nonrandom patterns of phylogenetic and trait dispersions were found across size classes at the 20 m × 20 m spatial scale (Figure 3; Figures S2–S9). The patterns generally tended toward overdispersion from clustering as size classes increased. However, some individual traits constantly showed clustered patterns across size classes (e.g., LPC; Figure S9).

**Figure 3:**
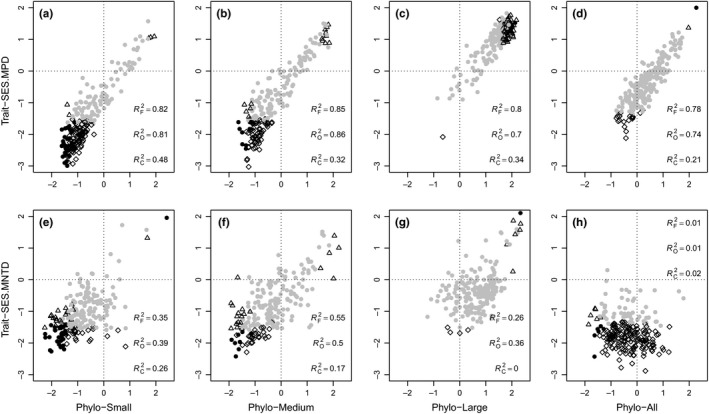
Distributions of phylogenetic (“Phylo”) and functional dispersions based on the combination of all traits (“Trait”) for two metrics (SES.MPD, SES.MNTD) across size classes at the spatial scale of 20 m × 20 m. “*R* ^2^.F/O/C” represents the adjusted regression coefficient *R* ^2^ between phylogenetic and functional dispersions when full, overdispersed, and clustered phylogenetic dispersions are included in our analyses, respectively. The closed gray circle indicates the random patterns for both phylogenetic and functional dispersions compared with 999 null communities. The open triangle represents a nonrandom pattern for only phylogenetic dispersion. The open rhombus indicates a nonrandom pattern for only functional dispersion, and the closed black circle indicates simultaneous nonrandom patterns for both phylogenetic and functional dispersions. Small: 1.0 cm ≤ dbh ≤ 5.0 cm; medium: 5.0 < dbh ≤ 10.0 cm; large: dbh > 10.0 cm; all: all trees dbh ≥ 1.0 cm regardless of whether it is a canopy species

### 3.2 The ability of phylogenetic distance to predict trait dispersion

We found some factors influenced the ability of phylogenetic distance to predict trait dispersion in this temperate forest. First, we found that overdispersed phylogenetic dispersion had a better relationship with trait dispersion at the 20 m × 20 m spatial scale than did clustered phylogenetic dispersion within each size class, and this result was conserved when we analyzed all traits together and when we analyzed individual traits (Figure 3; Figures S2–S9). Second, compared with SES.MNTD, the phylogenetic dispersion represented by SES.MPD was more tightly correlated with trait dispersion (Table 2; Table S1–S8). Moreover, the result was consistent when we controlled for purely spatial or jointly spatial and environmental effects, and when we assessed different spatial scales and size classes and analyzed all traits and eight individual traits (Table 2; Table S1–S8). Third, increasing spatial scales decreased the ability of SES.MNTD to predict trait dispersion, while it generally had no or weak effects on that for SES.MPD (Tables 2; Table S1–S8; especially for all traits, SLA, LA, and LT dispersions). However, this result was weakly influenced by spatial and environmental variables. Finally, the ability of phylogenetic distance to determine trait dispersion varied for different traits. The all traits, SLA, LA, and LT dispersions were well predicted by phylogenetic distance; the WD, SM, and *H*_max_ dispersions were poorly predicted by phylogenetic distance; and the LDMC and LPC generally were not predicted by phylogenetic distance (Table 2; Table S1–S8, Figures S10, S11). The result was also conserved when we controlled for purely spatial or jointly spatial and environmental effects, as well as when we assessed across spatial scales and size classes.

**Table 2:** Adjusted *R* ^2^ of the relationship between phylogenetic and functional dispersions based on the combination of all traits for two metrics (SES.MPD, SES.MNTD) across four spatial scales and size classes

All traits		10 m × 10 m		20 m × 20 m		30 m × 30 m		50 m × 50 m	
		SES.MPD	SES.MNTD	SES.MPD	SES.MNTD	SES.MPD	SES.MNTD	SES.MPD	SES.MNTD
All stems	Phylo	0.812	0.074	0.779	NA	0.740	NA	0.646	NA
	–ES	0.459	0.050	0.393	NA	0.382	NA	0.187	NA
	–S	0.484	0.042	0.391	NA	0.448	NA	0.413	NA
Small	Phylo	0.794	0.592	0.825	0.352	0.874	0.291	0.896	0.143
	–ES	0.480	0.366	0.390	0.113	0.334	0.162	0.326	0.016
	–S	0.490	0.401	0.419	0.125	0.338	0.159	0.446	0.016
Medium	Phylo	0.809	0.688	0.849	0.545	0.890	0.355	0.857	NA
	–ES	0.670	0.537	0.565	0.380	0.401	0.151	0.745	NA
	–S	0.694	0.559	0.536	0.390	0.431	0.145	0.656	NA
Large	Phylo	0.878	0.560	0.795	0.261	0.838	0.143	0.870	0.162
	–ES	0.584	0.443	0.296	0.112	0.278	0.060	0.200	0.120
	–S	0.618	0.446	0.333	0.126	0.396	0.069	0.648	0.125

Small: 1.0 cm ≤ dbh ≤ 5.0 cm; medium: 5.0 < dbh ≤ 10.0 cm; large: dbh > 10.0 cm; all stems: all trees dbh ≥ 1.0 cm regardless of whether it is a canopy species. “–ES/–S” indicates controlling for environmental and spatial effects or only spatial effects. “Phylo” indicates without considerations about environmental or spatial effects. “NA” indicates that the case could not perform variation partitioning analyses due to nonsignificant correlation between phylogenetic and trait dispersions.

## 4 DISCUSSION

In this study, we found that the phylogenetic signals presented at the species pool level were not preserved at the community level, which showed lower or higher signals than those at the species pool level. Partly due to the patterns in phylogenetic signals, the phylogenetic pattern, phylogenetic metric, spatial scale, and traits with different functions all influenced the ability of phylogenetic distance to predict trait dispersion in this temperate forest. Thus, we argued that the phylogenetic pattern might not necessarily be a poor proxy for trait dispersion in the temperate forest, but indeed depending on the phylogenetic patterns shown, metrics we used, spatial scales where our study conducted, and the traits we measured.

Our findings on the discordance of phylogenetic signals at the species pool and community levels questioned the prevalent tests reported in previous studies that focused on the species pool level (Swenson, Erickson, et al. 2012, Swenson, Stegen et al., 2012; Willis et al., 2010; Yang et al., 2014). Our result that the phylogenetic signals at the community level were generally lower than those at the species pool level in the studied temperate forest was consistent with the hypothesis proposed by Srivastava et al. (2012). However, we noted that the phylogenetic signals might also be higher in overdispersed communities. As a corollary, we found that the overdispersed pattern, rather than clustered pattern, was strongly correlated with trait dispersion because of the weaker phylogenetic signals detected in clustered communities. This result suggested that ecological strategies among co‐occurred closely related species tended to be dissimilar, which might be an important mechanism for species coexistence and community assembly (Chesson, 2000; HilleRisLambers, Adler, Harpole, Levine, & Mayfield, 2012; Keddy, 1992). Hierarchical assembly theory depicts that abiotic filtering acts on broader scales (e.g., β niche; Pickett & Bazzaz, 1978), which might constrain the evolution of traits to adapt to specific environments. At smaller scales (e.g., α niche; Pickett & Bazzaz, 1978), however, the trait evolution of co‐occurred closely related species was more labile to generate niche differentiation to enable coexistence (Silvertown, Dodd, et al. 2006, Silvertown, McConway, et al. 2006; but see Ackerly, Schwilk, & Webb, 2006). In addition, fitness differences likely caused either clustered phylogenetic dispersion or trait dispersion (Adler, Fajardo, Kleinhesselink, & Kraft, 2013; HilleRisLambers et al., 2012; Mayfield & Levine, 2010). Thus, both our findings and modern coexistence theory led us to rethink the processes carefully inferred from clustered phylogenetic patterns in previous studies.

We found that the ability of phylogenetic distance to determine trait dispersion decreased sharply when SES.MNTD was used rather than when SES.MPD was used in the temperate forest, and this result was conserved when we controlled for spatial and/or environmental variables. The result indicated that SES.MNTD might be more strongly influenced by the scale dependency of phylogenetic signals. There was an inconspicuous example from Liu et al. (2013), who also found this consistent result (see maximum height dispersion in their paper). Based on this point, we predicted and verified that the ability of SES.MNTD to determine trait dispersion decreased with an increase in spatial scale; when spatial scales increased, more species from the species pool were included and were more likely to co‐occur with their closely related species. For SES.MPD, the spatial scale had weak effects. However, the result was slightly influenced by spatial and environmental factors. This might be due to the increasing effects of environmental factors on trait dispersion at broader spatial scales (Cavender‐Bares et al., 2009; Swenson et al., 2007). As the ecological processes strongly depend on the spatial scale (Cavender‐Bares et al., 2009; Chalmandrier et al., 2017; Swenson et al., 2007), our study highlights the importance of the phylogenetic metric to correctly describe trait dispersion under the context of the spatial scale.

Corroborating our fourth prediction, phylogenetic dispersion was not a good proxy for all individual trait dispersions, although it predicted the multidimensional trait dispersion for SES.MPD well. This result was not surprising because dispersion patterns of individual traits might not be consistent within a given community, as shown in previous studies (Liu et al., 2013; Muscarella et al., 2016; Swenson & Enquist, 2009). The deeper reason might be that traits with contrasting functions or across different organs were usually decoupled (Baraloto et al., 2010; Laughlin & Wilson, 2014; Li et al., 2015); thus, the evolution of these traits might be independent along phylogenetic history. There were some questions remaining to be answered but important. For example, which trait axes were phylogenetically conserved across broader geographical and phylogenetic scales? Are those conserved traits or labile traits or both more important for species coexistence? In terms of the second question, if conserved traits were important, it would be safe to implement phylogenetic analyses. However, in some situations, labile traits among closely related species might be more important for niche differentiation. Adaptive radiation is a good example of this scenario; for example, many of Darwin's finches that inhabit an island display a diversification of their beaks to generate niche differentiation.

In our study of a temperate forest, the dispersions of LA, SLA, and LT with higher *K* values (>1) were best predicted by phylogenetic distance, whereas the dispersions of *H*_max_ and LDMC with lower *K* values (<1, but *p *<* *.05) were hardly predicted by phylogenetic distance. Therefore, based on our null model of a phylogenetic signal test, we argued that *p *<* *.05 did not guarantee the phylogenetic distance as a good proxy for trait dispersion. For two tropical studies, none of the traits had *K* values greater than one, although some still had *p *<* *.05 (Swenson, Stegen et al., 2012; Yang et al., 2014). In general, there are more species within genera or families in tropical forests than in temperate forests (i.e., tropical forests have a higher ratio of the number of species to genera or species to family; Table S9). Based on the hierarchical theory of phylogenetic signals (Silvertown, Dodd, et al. 2006, Silvertown, McConway, et al. 2006), increasing the number of species presented at a finer phylogenetic scale tends to decrease the phylogenetic signals. This may be the underlying cause for the observed inconsistencies in the relationship between phylogenetic and trait dispersions across temperate and tropical forests (Swenson, Erickson, et al. 2012, Swenson, Stegen et al., 2012; Yang et al., 2014).

Recently, phylogenetic approaches are still frequently being used worldwide across different ecosystems (Patrick & Stevens, 2016; Yan, Xie, Li, Holyoak, & Zhang, 2016; Zhu, Comita, Hubbell, Ma, & Shefferson, 2015). Our study showed that caution should be taken when inferring ecological mechanisms from phylogenetic patterns. Phylogenetic distance as a proxy of trait similarity worked well only in the situations we illustrated above in the temperate forest. More importantly, our results might be helpful in answering why phylogeny worked better than traits under some circumstances but not under other circumstances (Cadotte, Cavender‐Bares, Tilman, & Oakley, 2009; Cahill, Kembel, Lamb, & Keddy, 2008). Our study in the temperate forest partially tested the factors that influenced the relationship between phylogenetic distance and ecological differences, as suggested by Cadotte et al. (2017). However, our study was more focused on providing a guide for the appropriate application of phylogenetic patterns because the four factors we proposed might be usually encountered by community ecologists when they analyzed phylogenetic patterns. Further studies are needed to explore and test the framework of Cadotte et al. (2017). For example, the influence of intraspecific trait variation should be considered when evaluating the relationship between phylogenetic distance and ecological differences. In addition, our results concluded based on the study of a temperate forest might not be easily expanded to other ecosystems (e.g., tropical forests); thus, to generalize these conclusions, more studies should be conducted in different ecosystems.

## 5 CONCLUSIONS

In our 9‐ha old‐growth temperate forest dynamics plot, we found a discordance of phylogenetic signals between the species pool and community levels. More conserved signals were found within the overdispersed communities, whereas more labile signals were found within the clustered communities to maintain the coexistence among closely related species. The scale dependency of phylogenetic signals had a strong effect on the ability of phylogenetic distance to predict trait dispersion. Phylogenetic distance as a proxy of trait similarity might work when species are sampled randomly from a species pool for conserved traits. Based on this perspective, our study showed that the pattern of phylogenetic dispersion, phylogenetic metric, spatial scale, and functional dimensionality all potentially influenced the phylogenetic distance in the determination of trait dispersion. Based on our findings, we propose the careful application of phylogenetic‐based approaches. In addition, our results might increase our understanding about what factors may affect phylogenetic signals, as well as the relationship between phylogenetic diversity and ecosystem functioning.

## CONFLICTS OF INTEREST

The authors declare no conflict of interest.

## AUTHOR CONTRIBUTIONS

FJ and GZJ conceived the idea. FJ, YHX and HYC collected the soil, topological and trait data. FJ, YHX, and HYC analyzed the data. FJ and GZJ wrote the manuscript; other authors provided editorial advice.
