# Supplementary material for: What factors potentially influence the ability of phylogenetic distance to predict trait dispersion in a temperate forest?
Source: Ecol Evol. 2017 Dec 20;8(2):1107–16. doi: 10.1002/ece3.3691 (PMC5773330; doi:10.1002/ece3.3691)
Supplement: Supplementary file 1 [file ECE3-8-1107-s001.docx]

**What factors potentially influence the ability of phylogenetic distance to predict trait dispersion in a temperate forest?**

Feng Jiang ([jiangf951@126.com)^1^](mailto:jiangf951@126.com)1) Yanhan Xun ([violet1711@sina.cn)^1^](mailto:violet1711@sina.cn)1) Huiying Cai ([caihy0606@126.com)^1^](mailto:caihy0606@126.com)1) Guangze Jin ([taxus@126.com)^1*^](mailto:taxus@126.com)1*)

^1^ Center for Ecological Research, Northeast Forestry University, Harbin, China, 150040.

*Correspondence author: E-mail: [taxus@126.com](mailto:taxus@126.com); ORCID: [0000-0002-9852-0965](http://iauthor.cn/CN/0000-0002-9852-0965).

**Table S1.** Adjusted *R*^2^ of the relationship between phylogenetic and functional dispersions based on woody density for two metrics (SES.MPD, SES.MNTD) across four spatial scales and size classes.

| Woody density | | 10 m × 10 m | |  | 20 m × 20 m | |  | 30 m × 30 m | |  | 50 m × 50 m | |
| --- | --- | --- | --- | --- | --- | --- | --- | --- | --- | --- | --- | --- |
|  |  | SES.MPD | SES.MNTD |  | SES.MPD | SES.MNTD |  | SES.MPD | SES.MNTD |  | SES.MPD | SES.MNTD |
| All stems | Phylo | 0.468 | 0.066 |  | 0.382 | NA |  | 0.352 | NA |  | 0.204 | NA |
|  | -ES | 0.233 | 0.031 |  | 0.165 | NA |  | 0.156 | NA |  | 0.005 | NA |
|  | -S | 0.250 | 0.027 |  | 0.271 | NA |  | 0.137 | NA |  | 0.163 | NA |
| Small | Phylo | 0.218 | 0.050 |  | 0.204 | NA |  | 0.228 | NA |  | 0.193 | NA |
|  | -ES | 0.140 | 0.041 |  | 0.075 | NA |  | 0.029 | NA |  | 0.045 | NA |
|  | -S | 0.137 | 0.041 |  | 0.059 | NA |  | 0.056 | NA |  | 0.045 | NA |
| Medium | Phylo | 0.308 | 0.176 |  | 0.336 | 0.078 |  | 0.361 | 0.052 |  | 0.313 | NA |
|  | -ES | 0.232 | 0.123 |  | 0.101 | 0.013 |  | 0.087 | -0.002ns |  | 0.119 | NA |
|  | -S | 0.215 | 0.126 |  | 0.103 | 0.013 |  | 0.182 | -0.002ns |  | 0.293 | NA |
| Large | Phylo | 0.262 | 0.085 |  | 0.075 | NA |  | NA | NA |  | NA | NA |
|  | -ES | 0.172 | 0.071 |  | 0.055 | NA |  | NA | NA |  | NA | NA |
|  | -S | 0.173 | 0.065 |  | 0.067 | NA |  | NA | NA |  | NA | NA |

Small: 1.0 cm ≤ dbh ≤ 5.0 cm; Medium: 5.0 < dbh ≤ 10.0 cm; Large: dbh > 10.0 cm; All stems: all trees dbh ≥ 1.0 cm regardless of whether it is a canopy species. ‘-ES/-S’ indicates controlling for environmental and spatial effects or only spatial effects. ‘Phylo’ indicates without considerations about environmental or spatial effects. ‘NA’ indicates that the case could not perform variation partitioning analyses due to non-significant correlation between phylogenetic and trait dispersions.

**Table S2.** Adjusted *R*^2^ of the relationship between phylogenetic and functional dispersions based on seed mass for two metrics (SES.MPD, SES.MNTD) across four spatial scales and size classes.

| Seed mass | | 10 m × 10 m | |  | 20 m × 20 m | |  | 30 m × 30 m | |  | 50 m × 50 m | |
| --- | --- | --- | --- | --- | --- | --- | --- | --- | --- | --- | --- | --- |
|  |  | SES.MPD | SES.MNTD |  | SES.MPD | SES.MNTD |  | SES.MPD | SES.MNTD |  | SES.MPD | SES.MNTD |
| All stems | Phylo | 0.011 | 0.020 |  | NA | NA |  | NA | NA |  | NA | NA |
|  | -ES | 0.004 | 0.004 |  | NA | NA |  | NA | NA |  | NA | NA |
|  | -S | 0.004 | 0.003 |  | NA | NA |  | NA | NA |  | NA | NA |
| Small | Phylo | 0.153 | 0.089 |  | 0.171 | 0.034 |  | 0.203 | NA |  | 0.255 | NA |
|  | -ES | 0.063 | 0.024 |  | 0.038 | -0.003ns |  | 0.022 | NA |  | 0.054 | NA |
|  | -S | 0.064 | 0.031 |  | 0.040 | 0.001ns |  | 0.068 | NA |  | 0.028 | NA |
| Medium | Phylo | 0.107 | 0.078 |  | 0.051 | 0.016 |  | NA | NA |  | NA | NA |
|  | -ES | 0.086 | 0.059 |  | 0.036 | 0.008 |  | NA | NA |  | NA | NA |
|  | -S | 0.084 | 0.059 |  | 0.036 | 0.028 |  | NA | NA |  | NA | NA |
| Large | Phylo | 0.233 | 0.133 |  | 0.150 | 0.054 |  | 0.124 | NA |  | 0.140 | NA |
|  | -ES | 0.120 | 0.068 |  | 0.057 | 0.021 |  | 0.016 | NA |  | 0.110 | NA |
|  | -S | 0.129 | 0.079 |  | 0.093 | 0.018 |  | 0.095 | NA |  | 0.049 | NA |

Small: 1.0 cm ≤ dbh ≤ 5.0 cm; Medium: 5.0 < dbh ≤ 10.0 cm; Large: dbh > 10.0 cm; All stems: all trees dbh ≥ 1.0 cm regardless of whether it is a canopy species. ‘-ES/-S’ indicates controlling for environmental and spatial effects or only spatial effects. ‘Phylo’ indicates without considerations about environmental or spatial effects. ‘NA’ indicates that the case could not perform variation partitioning analyses due to non-significant correlation between phylogenetic and trait dispersions.

**Table S3.** Adjusted *R*^2^ of the relationship between phylogenetic and functional dispersions based on maximum height for two metrics (SES.MPD, SES.MNTD) across four spatial scales and size classes.

| Maximum height | | 10 m × 10 m | |  | 20 m × 20 m | |  | 30 m × 30 m | |  | 50 m × 50 m | |
| --- | --- | --- | --- | --- | --- | --- | --- | --- | --- | --- | --- | --- |
|  |  | SES.MPD | SES.MNTD |  | SES.MPD | SES.MNTD |  | SES.MPD | SES.MNTD |  | SES.MPD | SES.MNTD |
| All stems | Phylo | 0.019 | 0.029 |  | 0.015 | 0.058 |  | 0.071 | 0.044 |  | 0.177 | NA |
|  | -ES | 0.001ns | 0.012 |  | -0.002 | 0.007ns |  | -0.007 | 0.012ns |  | -0.008 | NA |
|  | -S | 0.002 | 0.012 |  | -0.002 | 0.012 |  | -0.004 | 0.012ns |  | -0.010 | NA |
| Small | Phylo | 0.168 | 0.171 |  | 0.113 | 0.149 |  | 0.103 | 0.136 |  | NA | NA |
|  | -ES | 0.071 | 0.074 |  | 0.037 | 0.049 |  | 0.057 | 0.003ns |  | NA | NA |
|  | -S | 0.068 | 0.076 |  | 0.045 | 0.042 |  | 0.024 | 0.046 |  | NA | NA |
| Medium | Phylo | 0.255 | 0.215 |  | 0.268 | 0.223 |  | 0.419 | 0.198 |  | 0.570 | 0.102 |
|  | -ES | 0.152 | 0.153 |  | 0.080 | 0.079 |  | 0.150 | 0.055 |  | 0.400 | 0.016ns |
|  | -S | 0.161 | 0.142 |  | 0.104 | 0.092 |  | 0.213 | 0.107 |  | 0.441 | 0.078 |
| Large | Phylo | 0.345 | 0.175 |  | 0.498 | 0.088 |  | 0.634 | 0.054 |  | 0.783 | NA |
|  | -ES | 0.151 | 0.101 |  | 0.073 | 0.024 |  | 0.048 | 0.006ns |  | 0.092 | NA |
|  | -S | 0.178 | 0.099 |  | 0.090 | 0.038 |  | 0.169 | 0.006ns |  | 0.418 | NA |

Small: 1.0 cm ≤ dbh ≤ 5.0 cm; Medium: 5.0 < dbh ≤ 10.0 cm; Large: dbh > 10.0 cm; All stems: all trees dbh ≥ 1.0 cm regardless of whether it is a canopy species. ‘-ES/-S’ indicates controlling for environmental and spatial effects or only spatial effects. ‘Phylo’ indicates without considerations about environmental or spatial effects. ‘NA’ indicates that the case could not perform variation partitioning analyses due to non-significant correlation between phylogenetic and trait dispersions.

**Table S4.** Adjusted *R*^2^ of the relationship between phylogenetic and functional dispersions based on specific leaf area for two metrics (SES.MPD, SES.MNTD) across four spatial scales and size classes.

| Specific leaf area | | 10 m × 10 m | |  | 20 m × 20 m | |  | 30 m × 30 m | |  | 50 m × 50 m | |
| --- | --- | --- | --- | --- | --- | --- | --- | --- | --- | --- | --- | --- |
|  |  | SES.MPD | SES.MNTD |  | SES.MPD | SES.MNTD |  | SES.MPD | SES.MNTD |  | SES.MPD | SES.MNTD |
| All stems | Phylo | 0.759 | 0.135 |  | 0.706 | 0.016 |  | 0.678 | 0.123 |  | 0.514 | 0.152 |
|  | -ES | 0.408 | 0.086 |  | 0.282 | 0.002ns |  | 0.328 | 0.026 |  | 0.366 | 0.004ns |
|  | -S | 0.447 | 0.076 |  | 0.381 | 0.000ns |  | 0.341 | 0.010ns |  | 0.384 | 0.050ns |
| Small | Phylo | 0.522 | 0.244 |  | 0.614 | 0.161 |  | 0.643 | 0.193 |  | 0.681 | 0.084ns |
|  | -ES | 0.299 | 0.160 |  | 0.239 | 0.074 |  | 0.125 | 0.065 |  | 0.142 | 0.115 |
|  | -S | 0.333 | 0.170 |  | 0.213 | 0.079 |  | 0.206 | 0.110 |  | 0.247 | 0.117 |
| Medium | Phylo | 0.624 | 0.436 |  | 0.731 | 0.310 |  | 0.786 | 0.247 |  | 0.796 | NA |
|  | -ES | 0.423 | 0.272 |  | 0.341 | 0.135 |  | 0.214 | 0.092 |  | 0.276 | NA |
|  | -S | 0.441 | 0.289 |  | 0.347 | 0.081 |  | 0.296 | 0.112 |  | 0.108 | NA |
| Large | Phylo | 0.749 | 0.307 |  | 0.718 | 0.046 |  | 0.749 | NA |  | 0.839 | 0.169 |
|  | -ES | 0.495 | 0.246 |  | 0.236 | 0.012 |  | 0.168 | NA |  | 0.335 | 0.049 |
|  | -S | 0.492 | 0.238 |  | 0.254 | 0.017 |  | 0.218 | NA |  | 0.289 | 0.237 |

Small: 1.0 cm ≤ dbh ≤ 5.0 cm; Medium: 5.0 < dbh ≤ 10.0 cm; Large: dbh > 10.0 cm; All stems: all trees dbh ≥ 1.0 cm regardless of whether it is a canopy species. ‘-ES/-S’ indicates controlling for environmental and spatial effects or only spatial effects. ‘Phylo’ indicates without considerations about environmental or spatial effects. ‘NA’ indicates that the case could not perform variation partitioning analyses due to non-significant correlation between phylogenetic and trait dispersions.

**Table S5.** Adjusted *R*^2^ of the relationship between phylogenetic and functional dispersions based on leaf area for two metrics (SES.MPD, SES.MNTD) across four spatial scales and size classes.

| Leaf area | | 10 m × 10 m | |  | 20 m × 20 m | |  | 30 m × 30 m | |  | 50 m × 50 m | |
| --- | --- | --- | --- | --- | --- | --- | --- | --- | --- | --- | --- | --- |
|  |  | SES.MPD | SES.MNTD |  | SES.MPD | SES.MNTD |  | SES.MPD | SES.MNTD |  | SES.MPD | SES.MNTD |
| All stems | Phylo | 0.952 | 0.351 |  | 0.961 | 0.164 |  | 0.967 | 0.183 |  | 0.959 | NA |
|  | -ES | 0.499 | 0.166 |  | 0.509 | 0.061 |  | 0.452 | 0.101 |  | 0.577 | NA |
|  | -S | 0.543 | 0.173 |  | 0.571 | 0.065 |  | 0.574 | 0.060 |  | 0.577 | NA |
| Small | Phylo | 0.805 | 0.497 |  | 0.889 | 0.323 |  | 0.923 | 0.212 |  | 0.955 | NA |
|  | -ES | 0.447 | 0.272 |  | 0.421 | 0.119 |  | 0.149 | 0.095 |  | 0.192 | NA |
|  | -S | 0.492 | 0.268 |  | 0.456 | 0.120 |  | 0.346 | 0.061 |  | 0.242 | NA |
| Medium | Phylo | 0.848 | 0.670 |  | 0.903 | 0.510 |  | 0.950 | 0.431 |  | 0.957 | NA |
|  | -ES | 0.638 | 0.496 |  | 0.468 | 0.286 |  | 0.471 | 0.233 |  | 0.553 | NA |
|  | -S | 0.678 | 0.538 |  | 0.453 | 0.273 |  | 0.402 | 0.202 |  | 0.273 | NA |
| Large | Phylo | 0.927 | 0.703 |  | 0.925 | 0.303 |  | 0.922 | 0.070 |  | 0.953 | NA |
|  | -ES | 0.604 | 0.520 |  | 0.388 | 0.161 |  | 0.227 | 0.060 |  | 0.153 | NA |
|  | -S | 0.644 | 0.510 |  | 0.421 | 0.169 |  | 0.269 | 0.052 |  | 0.444 | NA |

Small: 1.0 cm ≤ dbh ≤ 5.0 cm; Medium: 5.0 < dbh ≤ 10.0 cm; Large: dbh > 10.0 cm; All stems: all trees dbh ≥ 1.0 cm regardless of whether it is a canopy species. ‘-ES/-S’ indicates controlling for environmental and spatial effects or only spatial effects. ‘Phylo’ indicates without considerations about environmental or spatial effects. ‘NA’ indicates that the case could not perform variation partitioning analyses due to non-significant correlation between phylogenetic and trait dispersions.

**Table S6.** Adjusted *R*^2^ of the relationship between phylogenetic and functional dispersions based on leaf thickness for two metrics (SES.MPD, SES.MNTD) across four spatial scales and size classes.

| Leaf thickness | | 10 m × 10 m | |  | 20 m × 20 m | |  | 30 m × 30 m | |  | 50 m × 50 m | |
| --- | --- | --- | --- | --- | --- | --- | --- | --- | --- | --- | --- | --- |
|  |  | SES.MPD | SES.MNTD |  | SES.MPD | SES.MNTD |  | SES.MPD | SES.MNTD |  | SES.MPD | SES.MNTD |
| All stems | Phylo | 0.940 | 0.204 |  | 0.945 | NA |  | 0.946 | NA |  | 0.917 | NA |
|  | -ES | 0.520 | 0.112 |  | 0.552 | NA |  | 0.499 | NA |  | 0.515 | NA |
|  | -S | 0.559 | 0.085 |  | 0.561 | NA |  | 0.637 | NA |  | 0.515 | NA |
| Small | Phylo | 0.818 | 0.509 |  | 0.885 | 0.413 |  | 0.915 | 0.287 |  | 0.955 | NA |
|  | -ES | 0.516 | 0.325 |  | 0.342 | 0.189 |  | 0.249 | 0.151 |  | 0.174 | NA |
|  | -S | 0.522 | 0.346 |  | 0.514 | 0.203 |  | 0.339 | 0.139 |  | 0.212 | NA |
| Medium | Phylo | 0.825 | 0.619 |  | 0.890 | 0.467 |  | 0.933 | 0.397 |  | 0.947 | 0.119 |
|  | -ES | 0.629 | 0.437 |  | 0.428 | 0.190 |  | 0.424 | 0.140 |  | 0.246 | -0.005ns |
|  | -S | 0.664 | 0.466 |  | 0.431 | 0.193 |  | 0.508 | 0.164 |  | 0.238 | -0.008ns |
| Large | Phylo | 0.865 | 0.508 |  | 0.856 | 0.114 |  | 0.814 | NA |  | 0.885 | NA |
|  | -ES | 0.538 | 0.340 |  | 0.356 | 0.051 |  | 0.160 | NA |  | 0.211 | NA |
|  | -S | 0.576 | 0.339 |  | 0.369 | 0.058 |  | 0.231 | NA |  | 0.300 | NA |

Small: 1.0 cm ≤ dbh ≤ 5.0 cm; Medium: 5.0 < dbh ≤ 10.0 cm; Large: dbh > 10.0 cm; All stems: all trees dbh ≥ 1.0 cm regardless of whether it is a canopy species. ‘-ES/-S’ indicates controlling for environmental and spatial effects or only spatial effects. ‘Phylo’ indicates without considerations about environmental or spatial effects. ‘NA’ indicates that the case could not perform variation partitioning analyses due to non-significant correlation between phylogenetic and trait dispersions.

**Table S7.** Adjusted *R*^2^ of the relationship between phylogenetic and functional dispersions based on leaf dry matter content for two metrics (SES.MPD, SES.MNTD) across four spatial scales and size classes.

| Leaf dry matter content | | 10 m × 10 m | |  | 20 m × 20 m | |  | 30 m × 30 m | |  | 50 m × 50 m | |
| --- | --- | --- | --- | --- | --- | --- | --- | --- | --- | --- | --- | --- |
|  |  | SES.MPD | SES.MNTD |  | SES.MPD | SES.MNTD |  | SES.MPD | SES.MNTD |  | SES.MPD | SES.MNTD |
| All stems | Phylo | 0.003 | 0.025 |  | NA | NA |  | NA | 0.164 |  | NA | 0.216 |
|  | -ES | 0.004 | 0.021 |  | NA | NA |  | NA | 0.093 |  | NA | 0.040 |
|  | -S | 0.001ns | 0.016 |  | NA | NA |  | NA | 0.097 |  | NA | 0.071 |
| Small | Phylo | 0.145 | 0.159 |  | 0.204 | 0.070 |  | 0.322 | NA |  | 0.484 | NA |
|  | -ES | 0.074 | 0.094 |  | 0.037 | 0.018 |  | 0.029 | NA |  | 0.016ns | NA |
|  | -S | 0.086 | 0.102 |  | 0.058 | 0.026 |  | 0.029 | NA |  | 0.021ns | NA |
| Medium | Phylo | 0.077 | 0.085 |  | 0.069 | 0.027 |  | 0.050 | NA |  | NA | NA |
|  | -ES | 0.046 | 0.079 |  | 0.043 | 0.018 |  | 0.049 | NA |  | NA | NA |
|  | -S | 0.069 | 0.077 |  | 0.023 | 0.021 |  | 0.041 | NA |  | NA | NA |
| Large | Phylo | 0.094 | 0.046 |  | NA | NA |  | NA | NA |  | NA | 0.197 |
|  | -ES | 0.063 | 0.042 |  | NA | NA |  | NA | NA |  | NA | -0.008ns |
|  | -S | 0.068 | 0.035 |  | NA | NA |  | NA | NA |  | NA | 0.010ns |

Small: 1.0 cm ≤ dbh ≤ 5.0 cm; Medium: 5.0 < dbh ≤ 10.0 cm; Large: dbh > 10.0 cm; All stems: all trees dbh ≥ 1.0 cm regardless of whether it is a canopy species. ‘-ES/-S’ indicates controlling for environmental and spatial effects or only spatial effects. ‘Phylo’ indicates without considerations about environmental or spatial effects. ‘NA’ indicates that the case could not perform variation partitioning analyses due to non-significant correlation between phylogenetic and trait dispersions.

**Table S8.** Adjusted *R*^2^ of the relationship between phylogenetic and functional dispersions based on leaf phosphorus content for two metrics (SES.MPD, SES.MNTD) across four spatial scales and size classes.

| Leaf phosphorus content | | 10 m × 10 m | |  | 20 m × 20 m | |  | 30 m × 30 m | |  | 50 m × 50 m | |
| --- | --- | --- | --- | --- | --- | --- | --- | --- | --- | --- | --- | --- |
|  |  | SES.MPD | SES.MNTD |  | SES.MPD | SES.MNTD |  | SES.MPD | SES.MNTD |  | SES.MPD | SES.MNTD |
| All stems | Phylo | 0.083 | NA |  | 0.124 | NA |  | 0.172 | NA |  | 0.300 | NA |
|  | -ES | 0.016 | NA |  | 0.037 | NA |  | 0.046 | NA |  | 0.126 | NA |
|  | -S | 0.018 | NA |  | 0.039 | NA |  | 0.026 | NA |  | 0.251 | NA |
| Small | Phylo | NA | 0.007 |  | NA | NA |  | NA | NA |  | NA | 0.097 |
|  | -ES | NA | 0.003ns |  | NA | NA |  | NA | NA |  | NA | 0.071 |
|  | -S | NA | 0.004 |  | NA | NA |  | NA | NA |  | NA | 0.015ns |
| Medium | Phylo | 0.012 | NA |  | 0.060 | NA |  | 0.061 | NA |  | 0.088 | NA |
|  | -ES | 0.000ns | NA |  | 0.001ns | NA |  | -0.001ns | NA |  | 0.032ns | NA |
|  | -S | 0.000ns | NA |  | 0.005ns | NA |  | 0.005ns | NA |  | 0.002ns | NA |
| Large | Phylo | 0.136 | 0.097 |  | 0.185 | NA |  | 0.236 | NA |  | 0.272 | NA |
|  | -ES | 0.056 | 0.031 |  | 0.033 | NA |  | 0.004ns | NA |  | 0.020ns | NA |
|  | -S | 0.061 | 0.031 |  | 0.043 | NA |  | 0.092 | NA |  | 0.023ns | NA |

Small: 1.0 cm ≤ dbh ≤ 5.0 cm; Medium: 5.0 < dbh ≤ 10.0 cm; Large: dbh > 10.0 cm; All stems: all trees dbh ≥ 1.0 cm regardless of whether it is a canopy species. ‘-ES/-S’ indicates controlling for environmental and spatial effects or only spatial effects. ‘Phylo’ indicates without considerations about environmental or spatial effects. ‘NA’ indicates that the case could not perform variation partitioning analyses due to non-significant correlation between phylogenetic and trait dispersions.

**Table S9.** Some examples of the ratios of the number of species versus genera (S/G), species versus families (S/F) and genera versus families (G/F) across six temperate, subtropical and tropical forest dynamics plots (FDP).

| FDP | Species | Genera | Family | S/G | S/F | G/F | Year | Forest | Plot area |
| --- | --- | --- | --- | --- | --- | --- | --- | --- | --- |
| LS * | 41 | 31 | 19 | 1.323 | 2.158 | 1.632 | 2010 | temperate | 9 ha |
| WAB | 38 | 26 | 16 | 1.462 | 2.375 | 1.625 | 2008 | temperate | 25.2 ha |
| CBS | 52 | 32 | 18 | 1.625 | 2.889 | 1.778 | 2004 | temperate | 25 ha |
| GTS | 159 | 103 | 49 | 1.544 | 3.245 | 2.102 | 2005 | subtropical | 24 ha |
| BCI | 312 | 192 | 55 | 1.625 | 5.673 | 3.491 | --- | tropical | 50 ha |
| XSBN | 468 | 213 | 70 | 2.197 | 6.686 | 3.043 | 2007 | tropical | 20 ha |

LS: LiangShui FDP in our study; WAB: Wabikon FDP; CBS: ChangBaiShan FDP; GTS: GuTianShan FDP; BCI: Barro Colorado Island FDP; XSBN: XiShuangBanNa FDP.

**Figures**

**Fig. S1.** Molecular phylogeny of the 41 species included in our study. Scores on nodes indicate posterior probabilities based on 500 bootstraps.

**Fig. S2.** Distributions of phylogenetic (‘Phylo’) and functional dispersions based on woody density (WD) for two metrics (SES.MPD, SES.MNTD) across size classes at the spatial scale of 20 m × 20 m. ‘*R*^2^. F/O/C’ represent the adjusted regression coefficient *R*^2^ between phylogenetic and functional dispersions when full, overdispersed and clustered phylogenetic dispersions are included in our analyses, respectively. The closed gray circle indicates random patterns for both phylogenetic and functional dispersions compared with 999 null communities, the open triangle represents a non-random pattern for only phylogenetic dispersion, the open rhombus indicates non-random pattern for only functional dispersion and the closed black circle indicates simultaneous non-random patterns for both phylogenetic and functional dispersions. Small: 1.0 cm ≤ dbh ≤ 5.0 cm; Medium: 5.0 < dbh ≤ 10.0 cm; Large: dbh > 10.0 cm; All: all trees dbh ≥ 1.0 cm regardless of whether it is a canopy species.

**Fig. S3.** Distributions of phylogenetic (‘Phylo’) and functional dispersions based on seed mass (SM) for two metrics (SES.MPD, SES.MNTD) across size classes at the spatial scale of 20 m × 20 m. ‘*R*^2^. F/O/C’ represent the adjusted regression coefficient *R*^2^ between phylogenetic and functional dispersions when full, overdispersed and clustered phylogenetic dispersions are included in our analyses, respectively. The closed gray circle indicates random patterns for both phylogenetic and functional dispersions compared with 999 null communities, the open triangle represents a non-random pattern for only phylogenetic dispersion, the open rhombus indicates non-random pattern for only functional dispersion and the closed black circle indicates simultaneous non-random patterns for both phylogenetic and functional dispersions. Small: 1.0 cm ≤ dbh ≤ 5.0 cm; Medium: 5.0 < dbh ≤ 10.0 cm; Large: dbh > 10.0 cm; All: all trees dbh ≥ 1.0 cm regardless of whether it is a canopy species.

**Fig. S4.** Distributions of phylogenetic (‘Phylo’) and functional dispersions based on maximum height (*H*_max_) for two metrics (SES.MPD, SES.MNTD) across size classes at the spatial scale of 20 m × 20 m. ‘*R*^2^. F/O/C’ represent the adjusted regression coefficient *R*^2^ between phylogenetic and functional dispersions when full, overdispersed and clustered phylogenetic dispersions are included in our analyses, respectively. The closed grey circle indicates random patterns for both phylogenetic and functional dispersions compared with 999 null communities, the open triangle represents a non-random pattern for only phylogenetic dispersion, the open rhombus indicates non-random pattern for only functional dispersion and the closed black circle indicates simultaneous non-random patterns for both phylogenetic and functional dispersions. Small: 1.0 cm ≤ dbh ≤ 5.0 cm; Medium: 5.0 < dbh ≤ 10.0 cm; Large: dbh > 10.0 cm; All: all trees dbh ≥ 1.0 cm regardless of whether it is a canopy species.

**Fig. S5.** Distributions of phylogenetic (‘Phylo’) and functional dispersions based on specific leaf area (SLA) for two metrics (SES.MPD, SES.MNTD) across size classes at the spatial scale of 20 m × 20 m. ‘*R*^2^. F/O/C’ represent the adjusted regression coefficient *R*^2^ between phylogenetic and functional dispersions when full, overdispersed and clustered phylogenetic dispersions are included in our analyses, respectively. The closed gray circle indicates random patterns for both phylogenetic and functional dispersions compared with 999 null communities, the open triangle represents a non-random pattern for only phylogenetic dispersion, the open rhombus indicates non-random pattern for only functional dispersion and the closed black circle indicates simultaneous non-random patterns for both phylogenetic and functional dispersions. Small: 1.0 cm ≤ dbh ≤ 5.0 cm; Medium: 5.0 < dbh ≤ 10.0 cm; Large: dbh > 10.0 cm; All: all trees dbh ≥ 1.0 cm regardless of whether it is a canopy species.

**Fig. S6.** Distributions of phylogenetic (‘Phylo’) and functional dispersions based on leaf area (LA) for two metrics (SES.MPD, SES.MNTD) across size classes at the spatial scale of 20 m × 20 m. ‘*R*^2^. F/O/C’ represent the adjusted regression coefficient *R*^2^ between phylogenetic and functional dispersions when full, overdispersed and clustered phylogenetic dispersions are included in our analyses, respectively. The closed gray circle indicates random patterns for both phylogenetic and functional dispersions compared with 999 null communities, the open triangle represents a non-random pattern for only phylogenetic dispersion, the open rhombus indicates non-random pattern for only functional dispersion and the closed black circle indicates simultaneous non-random patterns for both phylogenetic and functional dispersions. Small: 1.0 cm ≤ dbh ≤ 5.0 cm; Medium: 5.0 < dbh ≤ 10.0 cm; Large: dbh > 10.0 cm; All: all trees dbh ≥ 1.0 cm regardless of whether it is a canopy species.

**Fig. S7.** Distributions of phylogenetic (‘Phylo’) and functional dispersions based on leaf thickness (LT) for two metrics (SES.MPD, SES.MNTD) across size classes at the spatial scale of 20 m × 20 m. ‘*R*^2^. F/O/C’ represent the adjusted regression coefficient *R*^2^ between phylogenetic and functional dispersions when full, overdispersed and clustered phylogenetic dispersions are included in our analyses, respectively. The closed gray circle indicates random patterns for both phylogenetic and functional dispersions compared with 999 null communities, the open triangle represents a non-random pattern for only phylogenetic dispersion, the open rhombus indicates non-random pattern for only functional dispersion and the closed black circle indicates simultaneous non-random patterns for both phylogenetic and functional dispersions. Small: 1.0 cm ≤ dbh ≤ 5.0 cm; Medium: 5.0 < dbh ≤ 10.0 cm; Large: dbh > 10.0 cm; All: all trees dbh ≥ 1.0 cm regardless of whether it is a canopy species.

**Fig. S8.** Distributions of phylogenetic (‘Phylo’) and functional dispersions based on leaf dry matter content (‘LDMC’) for two metrics (SES.MPD, SES.MNTD) across size classes at the spatial scale of 20 m × 20 m. ‘*R*^2^. F/O/C’ represent the adjusted regression coefficient *R*^2^ between phylogenetic and functional dispersions when full, overdispersed and clustered phylogenetic dispersions are included in our analyses, respectively. The closed gray circle indicates random patterns for both phylogenetic and functional dispersion compared with 999 null communities, the open triangle represents a non-random pattern for only phylogenetic dispersion, the open rhombus indicates non-random pattern for only functional dispersion and the closed black circle indicates simultaneous non-random patterns for both phylogenetic and functional dispersions. Small: 1.0 cm ≤ dbh ≤ 5.0 cm; Medium: 5.0 < dbh ≤ 10.0 cm; Large: dbh > 10.0 cm; All: all trees dbh ≥ 1.0 cm regardless of whether it is a canopy species.

**Fig. S9.** Distributions of phylogenetic (Phylo) and functional dispersions based on leaf phosphorus content (LPC) for two metrics (SES.MPD, SES.MNTD) across size classes at the spatial scale of 20 m × 20 m. ‘*R*^2^. F/O/C’ represent the adjusted regression coefficient *R*^2^ between phylogenetic and functional dispersions when full, overdispersed and clustered phylogenetic dispersions are included in our analyses, respectively. The closed gray circle indicates random patterns for both phylogenetic and functional dispersions compared with 999 null communities, the open triangle represents a non-random pattern for only phylogenetic dispersion, the open rhombus indicates non-random pattern for only functional dispersion and the closed black circle indicates simultaneous non-random patterns for both phylogenetic and functional dispersions. Small: 1.0 cm ≤ dbh ≤ 5.0 cm; Medium: 5.0 < dbh ≤ 10.0 cm; Large: dbh > 10.0 cm; All: all trees dbh ≥ 1.0 cm regardless of whether it is a canopy species.

**Fig. S10.** Principal component analyses (PCA) of phylogenetic (Phylo) and functional dispersions (including all traits (Trait), woody density (WD), seed mass (SM), maximum height (*H*_max_), specific leaf area (SLA), leaf area (LA), leaf thickness (LT), leaf dry matter content (LDMC) and leaf phosphorus content (LPC)) for two metrics (SES.MPD, SES.MNTD) when analysing all trees (dbh > 1cm) at the spatial scale of 20 m × 20 m.

**Fig. S11.** Principal component analyses (PCA) of phylogenetic (Phylo) and functional dispersions (including all traits (Trait), woody density (WD), seed mass (SM), maximum height (*H*_max_), specific leaf area (SLA), leaf area (LA), leaf thickness (LT), leaf dry matter content (LDMC) and leaf phosphorus content (LPC)) for two metrics (SES.MPD, SES.MNTD) when analysing small, medium and large size classes at the spatial scale of 20 m × 20 m. Small: 1.0 cm ≤ dbh ≤ 5.0 cm; Medium: 5.0 < dbh ≤ 10.0 cm; Large: dbh > 10.0 cm.**Fig. S1**

**Fig. S2.**

**Fig. S3.**

**Fig. S4.**

**Fig. S5.**

**Fig. S6.**

**Fig. S7.**

**Fig. S8.**

**Fig. S9.**

**Fig. S10.**

**Fig. S11.**
